# Supplementary material for: A history of avoidance does not impact extinction learning in male rats
Source: NPJ Sci Learn. 2024 Feb 24;9:11. doi: 10.1038/s41539-024-00223-z (PMC10894225; doi:10.1038/s41539-024-00223-z)
Supplement: Supplementary file 2 — Supplementary tables and figures [file 41539_2024_223_MOESM2_ESM.pdf]

Supplementary Figure 1

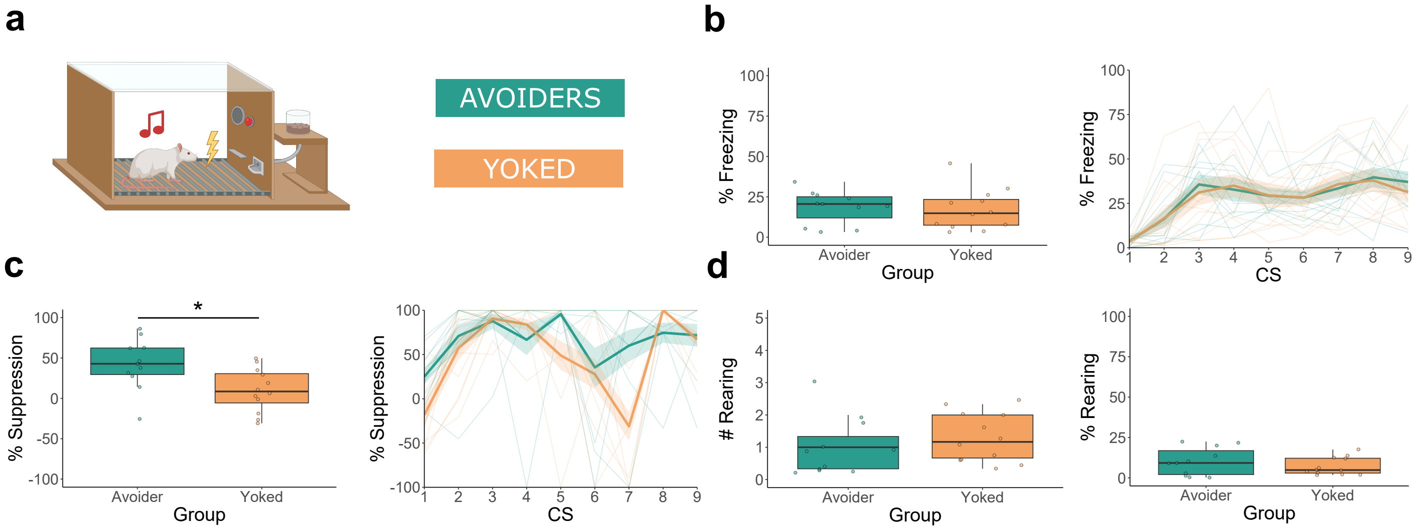

Supplementary Figure 1. Pavlovian acquisition results for Experiment 3. The box plots represent the average of the first 3 CSs. The bold lines in the trial-by-trial plots represent the mean and the surrounding shaded area the standard error of the mean. Results are expressed in % of time during CS presentations, except in rearing behavior where the first plot expresses the average number of rearing bouts during CS presentations. a. Graphical representation of the purely Pavlovian session which was identical for both groups. b. Both groups show similar freezing in Pavlovian acquisition. c. Rats assigned to be Avoiders in the subsequent avoidance learning phase suppress lever pressing more than their Yoked counterparts ($t(19.8) = 2.64$, $p = 0.016$, $d = 1.1$). d. The rate and duration of rearing is similar across groups in Pavlovian acquisition. *$p < 0.05$.

Supplementary Figure 2

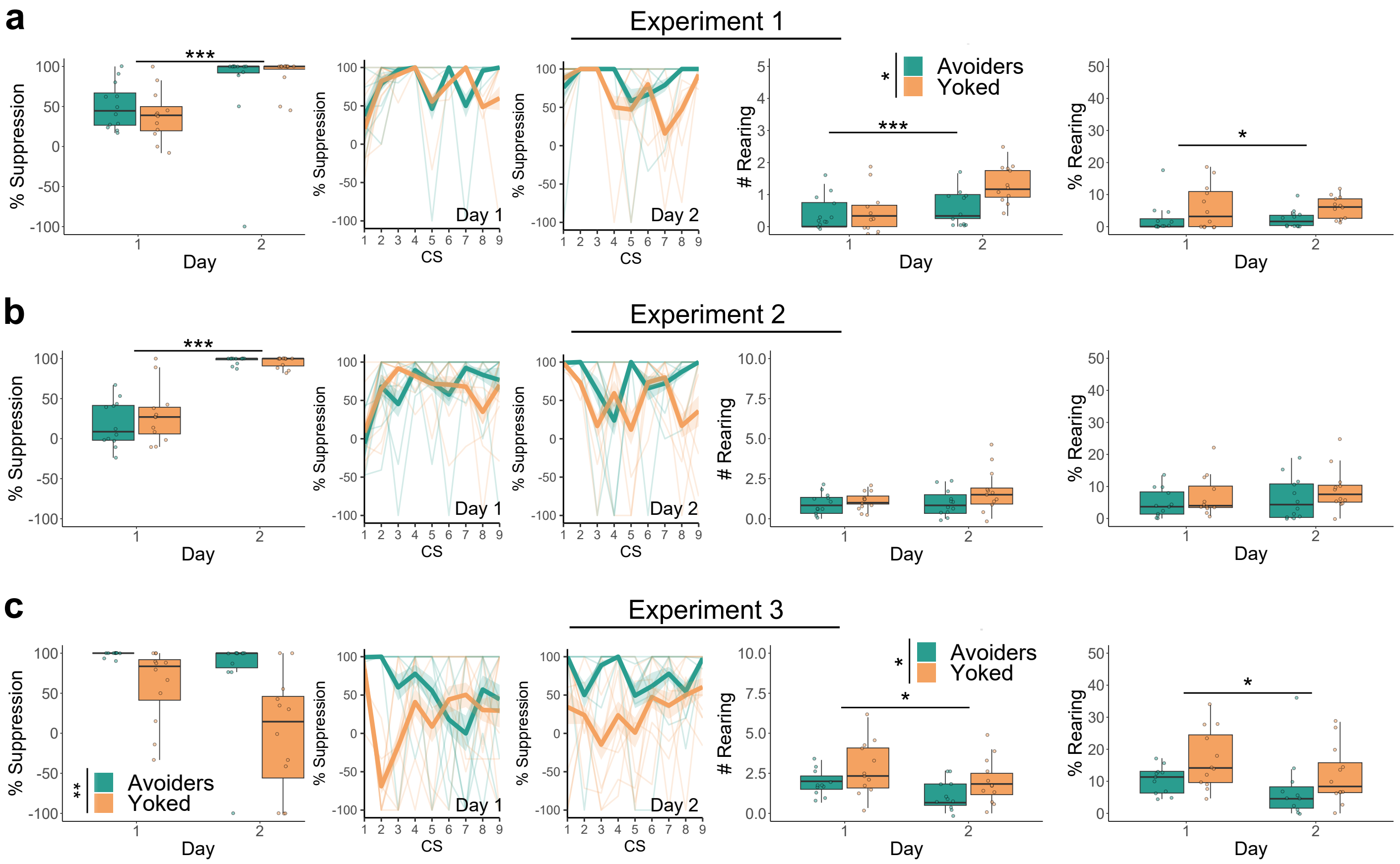

Supplementary Figure 2. Additional behaviors in avoidance training for Experiments 1 -3. The box plots represent the average of the first 3 CSs of every day. The bold lines in the trial-by-trial plots represent the mean and the surrounding shaded area the standard error of the mean. Results are expressed in % of time during CS presentations, except in rearing behavior where the first plot expresses the average number of rearing bouts during CS presentations. a. Both groups increased their suppression of lever pressing ($F(1, 22) = 18.32$, $p < 0.001$, $\eta p2 = 0.454$) and rearing events ($Q(1, 13.05) = 18.32$, $p < 0.001$, $\eta p2 = 0.281$) across training days in Experiment 1. However, Yoked rats showed increased rates ($Q(1, 13.43) = 4.95$, $p = 0.044$, $\eta p2 = 0.267$) and duration of rearing compared to Avoiders ($Q(1, 9.56) = 6.96$, $p = 0.026$, $\eta p2 = 0.234$). b. Both groups increased their suppression of lever pressing ($Q(1, 12.58) = 161.70$, $p < 0.001$, $\eta p2 = 0.854$) across training days in Experiment 2. c. Avoider rats showed increased suppression of lever pressing across avoidance training compared to their Yoked counterparts ($Q(1, 6.33) = 14.59$, $p = 0.008$, $\eta p2 = 0.38$), but Yoked rats reared more often than Avoiders across avoidance training sessions ($F(1, 21) = 4.68$, $p = 0.042$, $\eta p2 = 0.182$) in Experiment 3. A decrease in amount ($F(1, 21) = 6.66$, $p = 0.017$, $\eta p2 = 0.241$) and duration of rearing ($Q(1, 10.03) = 6.03$, $p = 0.034$, $\eta p2 = 0.105$) across avoidance training session is observed in Experiment 3. *$p < 0.05$, ** $p < 0.01$, ***$p < 0.001$.

Supplementary Figure 3

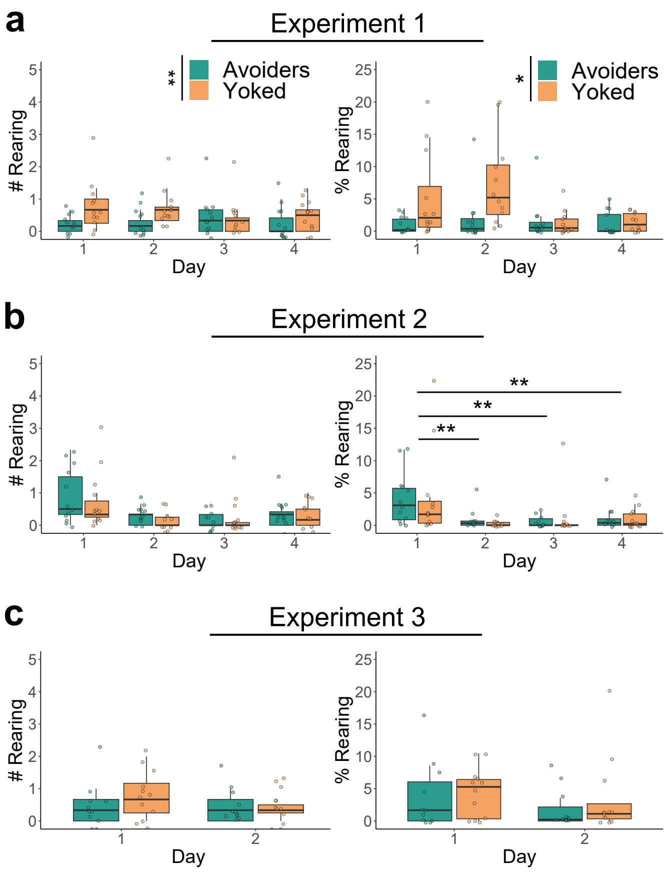

Supplementary Figure 3. Rearing events and duration in extinction training Experiments 1-3. The box plots represent the average of the first 3 CSs of every day. Results are expressed in % of time during CS presentations, except in rearing behavior where the first plot expresses the average number of rearing bouts during CS presentations. a. Across extinction training, Yoked rats present significantly higher rearing counts (Q(1, 14) = 10.15, p = 0.006, ηp2 = 0.219) and rearing duration (Q(1, 9.94) = 10.223, p = 0.01, ηp2 = 0.333) compared to Avoiders in Experiment 1. b. Both groups significantly reduce their rearing duration (Q(3, 9.88) = 3.94, p = 0.043, ηp2 = 0.276) across extinction training in Experiment 2. c. Rearing events and duration in Experiment 3 are not significantly different across group and training session. *p < 0.05, ** p < 0.01.

Supplementary Figure 4

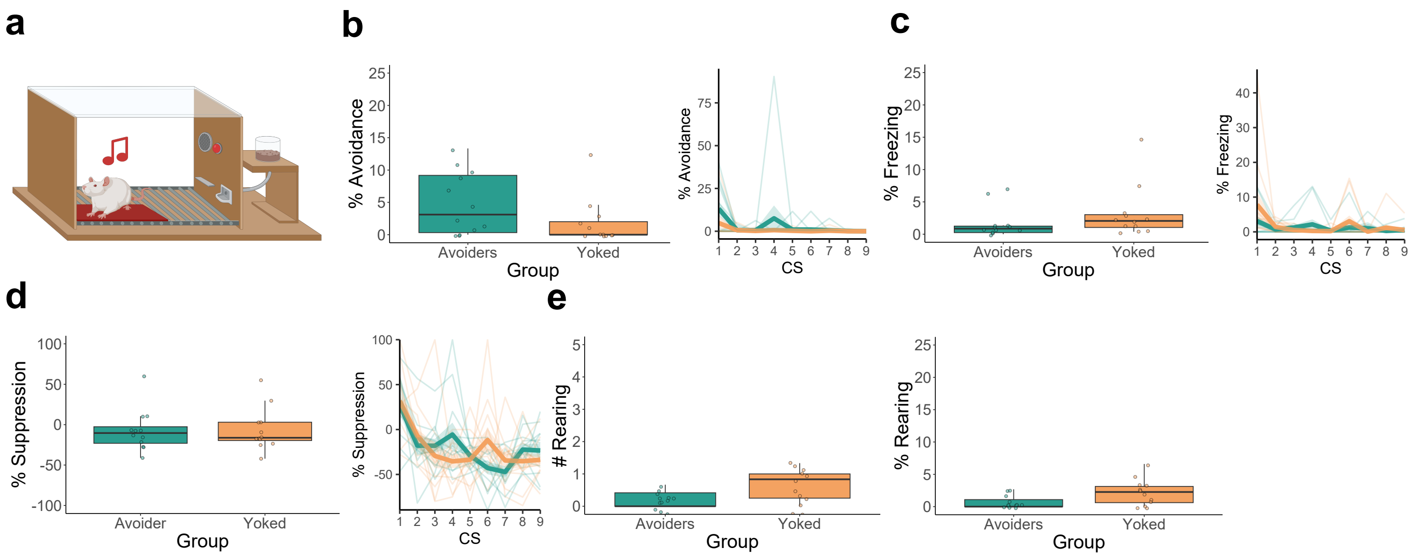

Supplementary Figure 4. Extinction with platform results for Experiment 1. The box plots represent the average of the first 3 CSs. The bold lines in the trial-by-trial plots represent the mean and the surrounding shaded area the standard error of the mean. Results are expressed in % of time during CS presentations, except in rearing behavior where the first plot expresses the average number of rearing bouts during CS presentations. a. Graphical representation of the extinction with platform session. b. Both groups show similar low levels of avoidance in the extinction session with platform. c. An interaction between group and block ($Q(2, 8.88) = 6.68$, $p = 0.017$, $\eta p2 = 0.046$) in freezing in the extinction session with platform. Further analyses were not significant after multiple-testing correction. d. Both groups similarly reduced their suppression of lever pressing over the three blocks ($Q(2, 12.11) = 6.89$, $p = 0.01$, : $\eta p2 = 0.331$). e. Both groups similarly reduced their amount ($Q(2, 11.93) = 7.77$, $p = 0.007$, $\eta p2 = 0.279$) and duration of rearing ($Q(2, 11.72) = 6.52$, $p = 0.012$, $\eta p2 = 0.215$) over the blocks.

Supplementary Figure 5

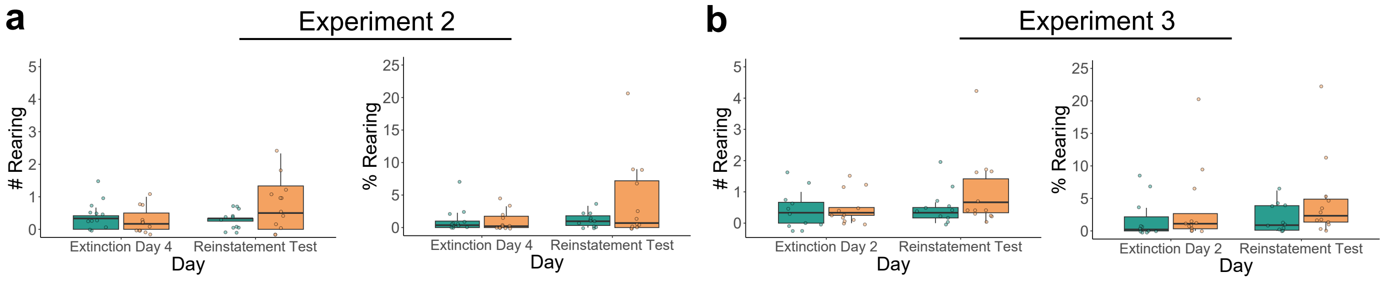

Supplementary Figure 5. Rearing events and duration in reinstatement test Experiments 2 and 3. The box plots represent the average of the first 3 CSs of the reinstatement test compared to the preceding extinction day. Results are expressed in % of time during CS presentations, except in rearing behavior where the first plot expresses the average number of rearing bouts during CS presentations. a. Both groups showed similar rearing behavior in Experiment 2. b Both groups showed similar rearing behavior in Experiment 3.

Supplementary Figure 6

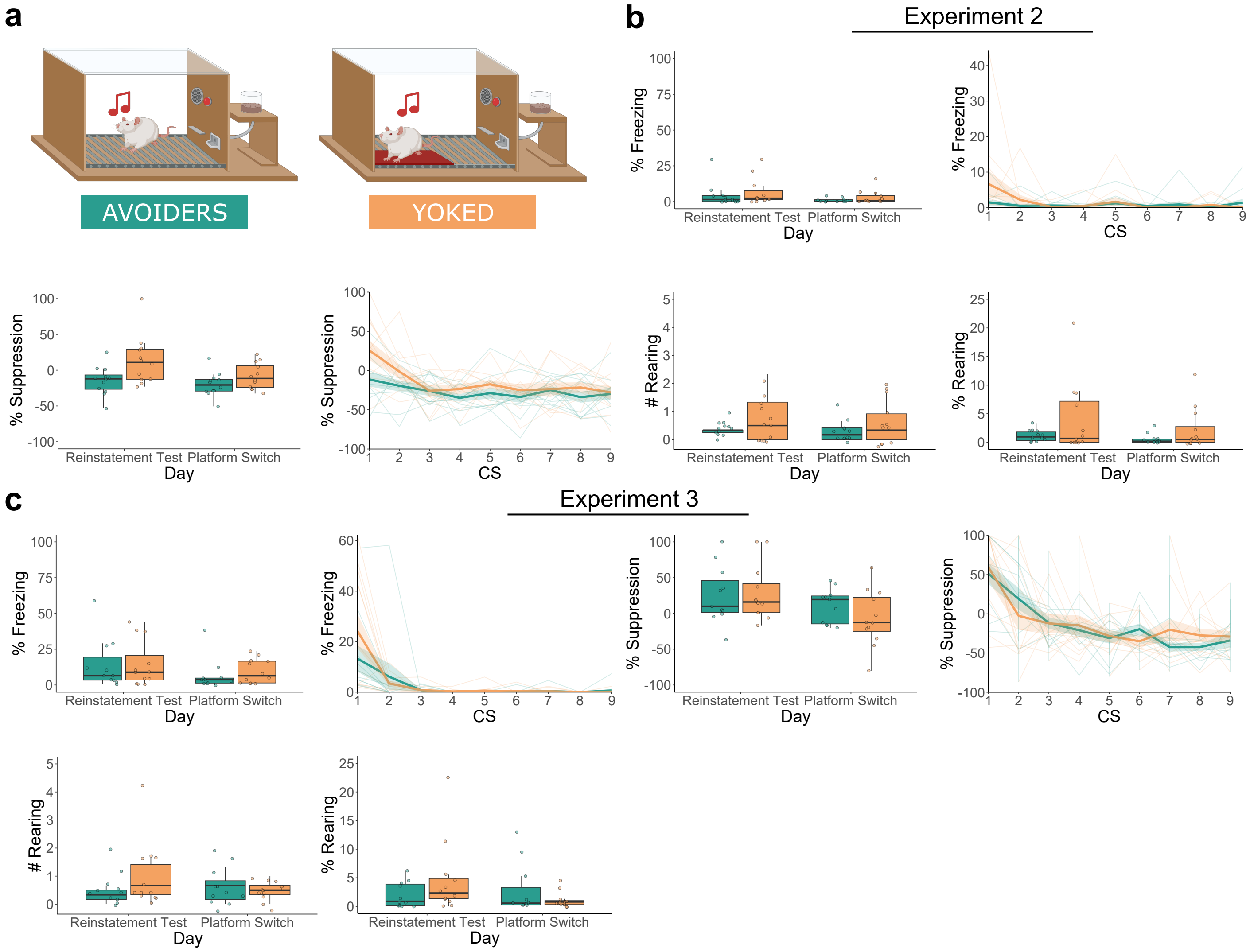

Supplementary Figure 6. Platform switch results for Experiment 2 and 3. The box plots represent the average of the first 3 CSs of the platform switch session compared to the preceding reinstatement test. The bold lines in the trial-by-trial plots represent the mean and the surrounding shaded area the standard error of the mean. Results are expressed in % of time during CS presentations, except in rearing behavior where the first plot expresses the average number of rearing bouts during CS presentations. a. Graphical representation of the platform switch session. b. Avoider and Yoked rats show similar freezing, suppression and rearing behavior in the platform switch session in Experiment 2. c. Both groups show similar freezing, suppression and rearing behavior in the platform switch session in Experiment 3.

Supplementary Figure 7

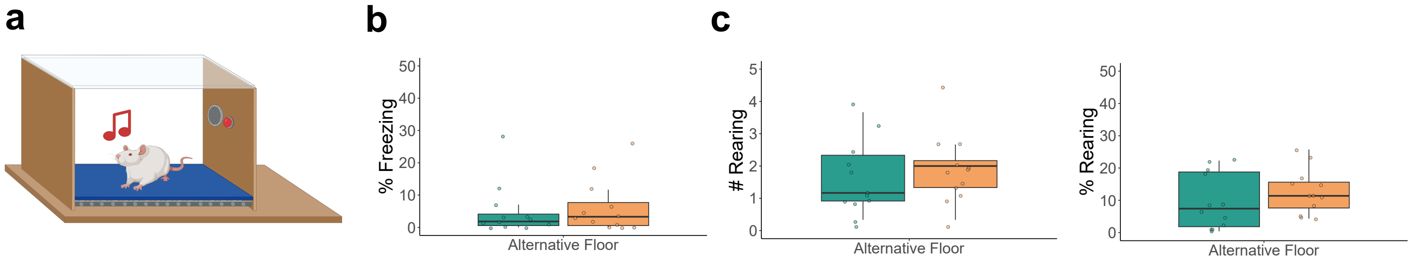

Supplementary Figure 7. Alternative floor results for Experiment 2. The box plots represent the average of the first 3 CSs. Results are expressed in % of time during CS presentations, except in rearing behavior where the first plot expresses the average number of rearing bouts during CS presentations. a. Graphical representation of the alternative floor session. b. Both groups show similar freezing in the alternative floor session in Experiment 2. c. Both groups showed similar amount and duration of rearing in the alternative floor session in Experiment 2.

Supplementary Figure 8

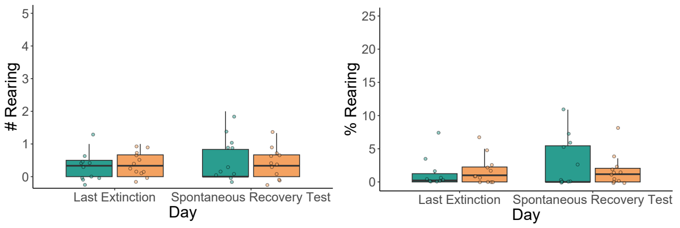

Supplementary Figure 8. The box plots represent the average of the first 3 CSs of the spontaneous recovery test compared to the preceding extinction session. The first plot expresses the number of rearing bouts during those CS presentations and in the second plot results are expressed in % of time during CS presentations. Both groups showed similar amount and duration of rearing during the spontaneous recovery test in Experiment 3.

Supplementary Tables

Supplementary Table 1. Pavlovian training

Experiment	Measure	Statistical Test	Result	Effect size
3	Freezing	T-test	t(20.6) = 0.298, p = 0.768	d = 0.124
	Suppression of lever pressing	T-test	t(19.8) = 2.64, **p = 0.016**	d = 1.1
	Rearing events	Mann Whitney U test	W = 50, p = 0.331	r = 0.209
	Rearing duration	T-test	t(16.7) = 1.02, p = 0.324	d = 0.428

Supplementary Table 2. Additional behaviors during avoidance training

Experiment	Measure	Statistical Test	Result	Effect size
1	Suppression of lever pressing	Mixed ANOVA	Group: F(1, 22) = 0, p = 0.954<br>Day: F(1, 22) = 18.32, **p < 0.001**<br>G*D: F(1, 22) = 1.65, p = 0.213	Group: $\eta_p^2 < 0.001$<br>Day: $\eta_p^2 = 0.454$<br>G*D: $\eta_p^2 = 0.07$
	Rearing events	Non-parametric mixed ANOVA	Group: Q(1, 13.43) = 4.95, **p = 0.044**<br>Day: Q(1, 13.05) = 18.32, **p < 0.001**<br>G*D: Q(1, 13.05) = 1.65, p = 0.213	Group: $\eta_p^2 = 0.267$<br>Day: $\eta_p^2 = 0.281$<br>G*D: $\eta_p^2 = 0.128$
	Rearing duration	Non-parametric mixed ANOVA	Group: Q(1, 9.56) = 6.96, **p = 0.026**<br>Day: Q(1, 9.35) = 0.55, p = 0.477<br>G*D: Q(1, 9.35) = 0.03, p = 0.868	Group: $\eta_p^2 = 0.234$<br>Day: $\eta_p^2 < 0.001$<br>G*D: $\eta_p^2 < 0.001$
2	Suppression of lever pressing	Non-parametric mixed ANOVA	Group: Q(1, 14) = 0.108, p = 0.747<br>Day: Q(1, 12.58) = 161.70, **p < 0.001**<br>G*D: Q(1, 12.58) = 0.40, p = 0.538	Group: $\eta_p^2 = 0.019$<br>Day: $\eta_p^2 = 0.854$<br>G*D: $\eta_p^2 = 0.045$
	Rearing events	Mixed ANOVA	Group: F(1, 22) = 3.65, p = 0.069<br>Day: F(1, 22) = 2.08, p = 0.163<br>G*D: F(1, 22) = 0.75, p = 0.396	Group: $\eta_p^2 = 0.142$<br>Day: $\eta_p^2 = 0.086$<br>G*D: $\eta_p^2 = 0.033$
	Rearing duration	Non-parametric mixed ANOVA	Group: Q(1, 12.45) = 1.45, p = 0.251<br>Day: Q(1, 13.99) = 0.61, p = 0.447<br>G*D: Q(1, 13.99) = 0.09, p = 0.773	Group: $\eta_p^2 = 0.086$<br>Day: $\eta_p^2 = 0.043$<br>G*D: $\eta_p^2 < 0.001$
3	Suppression of lever pressing	Non-parametric mixed ANOVA	Group: Q(1, 6.33) = 14.59, **p = 0.008**<br>Day: Q(1, 6.33) = 5.75, p = 0.051	Group: $\eta_p^2 = 0.38$<br>Day: $\eta_p^2 = 0.262$

			G*D: Q(1, 6.33) = 4.33, p = 0.08	G*D: η_p^2 = 0.073
	Rearing events	Mixed ANOVA	Group: F(1, 21) = 4.68, **p = 0.042**<br>Day: F(1, 21) = 6.66, **p = 0.017**<br>G*D: F(1, 21) = 0, p = 0.984	Group: η_p^2 = 0.182<br>Day: η_p^2 = 0.241<br>G*D: η_p^2 < 0.001
	Rearing duration	Non-parametric mixed ANOVA	Group: Q(1, 8.76) = 4.49, p = 0.064<br>Day: Q(1, 10.03) = 6.03, **p = 0.034**<br>G*D: Q(1, 10.03) = 0, p = 0.981	Group: η_p^2 = 0.163<br>Day: η_p^2 = 0.105<br>G*D: η_p^2 = 0.01

Supplementary Table 3. Rearing during extinction training

Experiment	Measure	Statistical Test	Result	Effect size
1	Rearing events	Non-parametric mixed ANOVA	Group: Q(1, 14) = 10.15, **p = 0.006**<br>Day: Q(3, 10.42) = 0.283, p = 0.8363<br>G*D: Q(3, 10.42) = 1.77, p = 0.213	Group: η_p^2 = 0.219<br>Day: η_p^2 = 0.014<br>G*D: η_p^2 = 0.061
	Rearing duration	Non-parametric mixed ANOVA	Group: Q(1, 9.94) = 10.223, **p = 0.01**<br>Day: Q(3, 7.74) = 2.13, p = 0.176<br>G*D: Q(3, 7.74) = 2.09, p = 0.182	Group: η_p^2 = 0.333<br>Day: η_p^2 = 0.135<br>G*D: η_p^2 = 0.113
2	Rearing events	Non-parametric mixed ANOVA	Group: Q(1, 13.65) = 1.84, p = 0.197<br>Day: Q(3, 10.88) = 2.28, p = 0.137<br>G*D: Q(3, 10.88) = 0.24, p = 0.869	Group: η_p^2 = 0.008<br>Day: η_p^2 = 0.225<br>G*D: η_p^2 = 0.024
	Rearing duration	Non-parametric mixed ANOVA	Group: Q(1, 13.68) = 1.52, p = 0.239<br>Day: Q(3, 9.88) = 3.94, **p = 0.043**<br>G*D: Q(3, 9.88) = 0.45, p = 0.725	Group: η_p^2 < 0.001<br>Day: η_p^2 = 0.276<br>G*D: η_p^2 = 0.008
	Rearing duration	Pairwise comparisons with Wilcoxon rank sum test with Bonferroni correction	Day 1-2: **p = 0.002**<br>Day 1-3: **p = 0.002**<br>Day 2-3: p = 1<br>Day 1-4: **p = 0.036**<br>Day 2-4: p = 1<br>Day 3-4: p = 1	
3	Rearing events	Non-parametric mixed ANOVA	Group: Q(1, 9.30) = 1.07, p = 0.327<br>Day: Q(1, 12) = 2.64, p = 0.130<br>G*D: Q(1, 12) = 1.58, p = 0.232	Group: η_p^2 = 0.034<br>Day: η_p^2 = 0.122<br>G*D: η_p^2 = 0.062
	Rearing duration	Non-parametric mixed ANOVA	Group: Q(1, 11.8) = 0.83, p = 0.382<br>Day: Q(1, 11.08) = 3.81, p = 0.077	Group: η_p^2 = 0.028<br>Day: η_p^2 = 0.059

			G*D: Q(1, 11.08) = 0.19, p = 0.667	G*D: $\eta_p^2 = 0.007$

Supplementary Table 4. Extinction with platform in Experiment 1

Experiment	Measure	Statistical Test	Result	Effect size
1	Avoidance	Non-parametric mixed ANOVA	Group: Q(1, 7.69) = 2.36, p = 0.164<br>Block: Q(2, 7.18) = 3.59, p = 0.083<br>G*B: Q(2, 7.18) = 1.68, p = 0.252	Group: $\eta_p^2 = 0.116$<br>Block: $\eta_p^2 = 0.143$<br>G*B: $\eta_p^2 = 0.04$
	Freezing	Non-parametric mixed ANOVA	Group: Q(1, 13) = 2.7, p = 0.124<br>Block: Q(2, 8.88) = 10.96, **p = 0.004**<br>G*B: Q(2, 8.88) = 6.68, **p = 0.017**	Group: $\eta_p^2 = 0.021$<br>Block: $\eta_p^2 = 0.169$<br>G*B: $\eta_p^2 = 0.046$
	Freezing	Simple effects by day: Wilcoxon signed ranks test	Block 1 – Group: V= 39.5, p = 0.064<br>Block 2 – Group: V= 60.5, p = 0.497<br>Block 3 – Group: V= 92.5, p = 0.217	Block 1 – Group: r = 0.384<br>Block 2 – Group: r = 0.145<br>Block 3 – Group: r = 0.258
	Suppression of lever pressing	Non-parametric mixed ANOVA	Group: Q(1, 13.2) = 0.19, p = 0.664<br>Block: Q(2, 12.11) = 6.89, **p = 0.01**<br>G*B: Q(2, 12.11) = 0.19, p = 0.829	Group: $\eta_p^2 = 0.003$<br>Block: $\eta_p^2 = 0.331$<br>G*B: $\eta_p^2 = 0.003$
	Suppression of lever pressing	Pairwise comparisons with Wilcoxon rank sum test with Bonferroni correction	Block 1-2: **p = 0.003**<br>Block 1-3: **p < 0.001**<br>Block 2-3: p = 1	
	Rearing events	Non-parametric mixed ANOVA	Group: Q(1, 13.16) = 3.06, p = 0.103<br>Block: Q(2, 11.93) = 7.77, **p = 0.007**<br>G*B: Q(2, 11.93) = 2.76, p = 0.103	Group: $\eta_p^2 = 0.144$<br>Block: $\eta_p^2 = 0.279$<br>G*B: $\eta_p^2 = 0.185$
	Rearing events	Pairwise comparisons with Wilcoxon rank sum test with Bonferroni correction	Block 1-2: **p = 0.005**<br>Block 1-3: p = 0.335<br>Block 2-3: p = 0.11	
	Rearing duration	Non-parametric mixed ANOVA	Group: Q(1, 12.17) = 3.1, p = 0.103<br>Block: Q(2, 11.72) = 6.52, **p = 0.012**<br>G*B: Q(2, 11.72) = 2.53, p = 0.122	Group: $\eta_p^2 = 0.082$<br>Block: $\eta_p^2 = 0.215$<br>G*B: $\eta_p^2 = 0.147$
	Rearing duration	Pairwise comparisons with Wilcoxon rank sum test with Bonferroni correction	Block 1-2: p = 0.006<br>Block 1-3: p = 0.579<br>Block 2-3: p = 0.098	

Supplementary Table 5. Rearing during the reinstatement test

Experiment	Measure	Statistical Test	Result	Effect size
2	Rearing events	Non-parametric mixed ANOVA	Group: Q(1, 8.89) = 0.36, p = 0.565<br>Day: Q(1, 10.84) = 1.68, p = 0.222<br>G*D: Q(1, 10.84) = 1.68, p = 0.222	Group: η_p^2 = 0.055<br>Day: η_p^2 = 0.098<br>G*D: η_p^2 = 0.162
	Rearing duration	Non-parametric mixed ANOVA	Group: Q(1, 8.23) = 0.48, p = 0.508<br>Day: Q(1, 8.32) = 2.02, p = 0.19<br>G*D: Q(1, 8.32) = 0.73, p = 0.418	Group: η_p^2 = 0.081<br>Day: η_p^2 = 0.105<br>G*D: η_p^2 = 0.105
3	Rearing events	Non-parametric mixed ANOVA	Group: Q(1, 9.06) = 1.23, p = 0.296<br>Day: Q(1, 11.67) = 3.05, p = 0.107<br>G*D: Q(1, 11.67) = 1.83, p = 0.201	Group: η_p^2 = 0.051<br>Day: η_p^2 = 0.120<br>G*D: η_p^2 = 0.079
	Rearing duration	Non-parametric mixed ANOVA	Group: Q(1, 11.07) = 1.04, p = 0.329<br>Day: Q(1, 11.97) = 1.44, p = 0.253<br>G*D: Q(1, 11.97) = 0.05, p = 0.823	Group: η_p^2 = 0.098<br>Day: η_p^2 = 0.009<br>G*D: η_p^2 = 0.006

Supplementary Table 6. Platform switch session

Experiment	Measure	Statistical Test	Result	Effect size
2	Freezing	Mann Whitney U test	W = 45.5, p = 0.119	r = 0.324
	Suppression of lever pressing	T-test	t(21.8) = -1.63, p = 0.117	d = -0.67
	Rearing events	Mann Whitney U test	W = 56, p = 0.346	r = 0.199
	Rearing duration	Mann Whitney U test	W = 52.5 p = 0.255	r = 0.239
3	Freezing	Mann Whitney U test	W = 50, p = 0.34	r = 0.205
	Suppression of lever pressing	T-test	t(18.2) = 1.48, p = 0.157	d = 0.603
	Rearing events	Mann Whitney U test	W = 72, p = 0.726	r = 0.079
	Rearing duration	Mann Whitney U test	W = 72, p = 0.733	r = 0.077

Supplementary Table 7. Alternative floor session

Experiment	Measure	Statistical Test	Result	Effect size
2	Freezing	Mann Whitney U test	W = 65, p = 0.706	r = 0.083
	Rearing events	T-test	t(22) = -0.810, p = 0.426	d = -0.173

	Rearing duration	Mann Whitney U test	W = 50, p = 0.214	r = 0.259

Supplementary Table 8. Rearing during the spontaneous recovery test

Experiment	Measure	Statistical Test	Result	Effect size
3	Rearing events	Non-parametric mixed ANOVA	Group: Q(1, 10.43) = 0.07, p = 0.796<br>Day: Q(1, 10.41) = 0.12, p = 0.74<br>G*D: Q(1, 10.41) = 0.12, p = 0.74	Group: $\eta_p^2 < 0.001$<br>Day: $\eta_p^2 = 0.057$<br>G*D: $\eta_p^2 = 0.034$
	Rearing duration	Non-parametric mixed ANOVA	Group: Q(1, 8.10) = 0.03, p = 0.866<br>Day: Q(1, 8.67) = 0.88, p = 0.373<br>G*D: Q(1, 8.67) = 0.92, p = 0.364	Group: $\eta_p^2 = 0.009$<br>Day: $\eta_p^2 = 0.049$<br>G*D: $\eta_p^2 = 0.01$
